# Responses of annual herb plant community characteristics to increased precipitation and reduced wind velocity in semiarid sandy grassland

**DOI:** 10.1002/ece3.5585

**Published:** 2019-09-05

**Authors:** Shan‐Shan Sun, Xin‐Ping Liu, Yu‐Hui He, Shui‐Lian Wei, La‐Mei Zhang, Peng Lv, Chelmeg Bao, Ming‐Ming Wang, Li Cheng

**Affiliations:** ^1^ Naiman Desertification Research Station Northwest Institute of Eco‐Environment and Resources Chinese Academy of Sciences Lanzhou China; ^2^ University of Chinese Academy of Sciences Beijing China; ^3^ Urat Desert‐grassland Research Station Northwest Institute of Eco‐Environment and Resources Chinese Academy of Sciences Lanzhou China; ^4^ Beijing ZTRC Environmental Protection Science &Technology Co., Ltd Beijing China; ^5^ Forestry and Grassland Service Center in Tongwei County Dingxi China

**Keywords:** community characteristic, Horqin Sandy Land, precipitation, wind velocity

## Abstract

Changes in precipitation regimes and wind velocity tend to alter structure and composition of the annual herb plant community, with consequent effects on ecological functioning and biodiversity maintenance. We examined the effects of increased precipitation and reduced wind velocity on annual herb plant community characteristics via a manipulative experiment from the middle of April to middle of August, 2016. There was significant increment in species richness with increased precipitation from June to August, and there were interactive effects between increased precipitation and reduced wind velocity especially in June and the end of July. From June to August, increased precipitation, reduced wind velocity as well as their interaction stimulated sandy plant community development. There was considerable elevation in plant coverage with increased precipitation, and also there was an interactive effect of increased precipitation with 20% reduced wind velocity. However, reduced wind velocity caused more significant stimulation (*p* < .01) in plant height. Moreover, dominant plants, *Salsola collina*, *Bassia dasyphylla*, and *Setaria viridis*, contributed equally to the elevated community coverage with increased precipitation, whereas *S. collina* occupied a much larger proportion on the augment of community height compared with the other two species under the increased precipitation and reduced wind velocity. Elevated Shannon–Wiener index was detected with increased precipitation in June and July. Furthermore, increased precipitation and reduced wind velocity enhanced aboveground and belowground biomass, respectively. These species traits‐in structuring and composing plant community were suggested to be conducive to deep understanding the plant functioning and dynamics under global changing precipitation regimes and atmospheric wind velocity scenarios.

## Graphical abstract

We evaluated the responses of annual herb plant community characteristic to increased precipitation and reduced wind velocity with the manipulative experiment, we acknowledge that global changing precipitation regimes and wind velocity are likely to alter annual herb plant community structure and composition, then consequently affecting the biodiversity and ecosystem function, investigation on this will be pretty valuable and meaningful to understand annual herb plant functional mechanism and project vegetation dynamics in response to future global climate changes

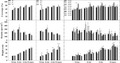

## 1 INTRODUCTION

Anthropogenic has intensified the globally and regionally environmental changes, including the increased precipitation frequency and intensity, as well as the extreme precipitation events (IPCC, 2007, 2013), with no spatial or temporal consistency (Walther et al., 2002; Fay, Carlisle, Knapp, Blair, & Collins, 2000). Changing climate will indisputably alter plant distribution and biological productivity (Wu, Dijkstra, Koch, Peñuelas, & Hungate, 2011; Harte & Shaw, 1995; Peter, 2009; Sternberg, Brown, Masters, & Clarke, 1999). In arid and semiarid regions, atmospheric wind flowing can also alter the precipitation frequency and intensity (Rosenfeld, Rudich, & Lahav, 2001). There was a decreased trend in the wind velocity from 1980s to the year of 2008 (Wang, Li, Dong, & Xia, 2008). Although there were few studies in the wind disturbance, it possibly has become the most critical disturbance in impacting the plant seed propagation, community composition, and dynamics. In conjunction with water availability, changing atmospheric wind flowing will have significant effects on the plant community structure, composition, and function. Though the response of plant community structure and composition to the individual changing environment factor has been substantially investigated (DeLucia et al., 1999; Lavorel & Garnier, 2002; Rundel, 1997), there is relatively little research exploring the two interactive effects of changing precipitation regimes and atmospheric wind activity on the community characteristics of the annual herb plant in arid and semiarid region. The occurrence of changing precipitation and wind activity speed is not unusual in arid and semiarid regions. These two factors were determinants of herbaceous plant community that could alter plant productivity and correlate with ecosystems functioning.

As an importantly limited factor, water availability has caused a range of changes in plant community structure and composition in arid and semiarid regions, including the wild species traits (Camille & Hanley, 2015), plant growth, and biomass (Wu et al., 2011). The ecosystem productivity of different plant functional types had a relationship with precipitation seasonal distribution in water‐limiting regions (Abraha et al., 2016; Reichmann & Sala, 2014), and the species richness was closely correlated with soil moisture (Yang et al., 2011, 2015). Moreover, it has also been clearly demonstrated that water availability, specifically the seasonal precipitation from January to July, definitely mediates plant community structure and composition in arid and semiarid regions (Chao et al., 2018; Wu et al., 2011). Furthermore, the effects of changing climate especially in precipitation regimes on life patterns and community evenness of dominant plants possibly alter plant community characteristics (Kardol et al., 2010). The research on discretely extreme precipitation events of global climate change was relatively rare (Jentsch, Kreyling, & Beierkuhnlein, 2007; Smith, 2011), so we cannot well learn the ecological response of plant community to the extreme precipitation events in water‐deficient areas (Hoover, Knapp, & Smith, 2014), where the ecosystem is vulnerable to the environmental variation (Wang et al., 2018).

The reduced atmospheric wind activity may alter plant growth pattern under changing climate condition (Buckley, 1987). Though lacking investigations, wind disturbance is likely to be an important factor to influence the structure and composition of plant community (Allen, Thapa, Arevalo, & Palmer, 2012; Peterson, 2000). The moderate wind blowing can promote both of the stem density and regeneration of forest plants (Shorohova, Fedorchuk, Kuznetsova, & Shvedova, 2008). Moreover, the spatial interaction between changing wind activity and other climatic factor can impact the aboveground biomass and species composition in the northern forest ecosystems (Scheller & Mladenoff, 2005). Although the changes in global precipitation regimes are unquestioned, the interactive mechanism of changing atmospheric wind and precipitation remains uncertain, the responses of plant community characteristic to both of that were not acknowledged (Miguel, Lavariega, & Moreno, 2017; Sydeman et al., 2014). Given that precipitation magnitude is critical to impact the plant growth (Serreze et al., 2000; Yang et al., 2016), and taking the atmospheric wind into consideration, the precipitation increase and wind velocity reduction, as well as the interaction of the two factors will complicatedly affect structure and composition of the annual herb plant community, with consequent impacts on ecosystem functional mechanism in arid and semiarid regions.

Many herbaceous plants grow on the grassland, feeding a large proportion of livestock worldwide (Morgan et al., 2011; Suttie et al., 2005). The semiarid region, Horqin Sandy Land, is one of the four major sandy areas in China, located in the eastern Inner Mongolia (Liu, He, Zhang, et al., 2015; Liu, He, Zhao, et al., 2015; Zhao et al., 2010). The precipitation regimes and atmosphere wind velocity have changed with the global climate change in this region; this regional ecosystem was also susceptible to changing environment (Christensen, Coughenour, Ellis, & Chen, 2004; Niu et al., 2008; Zhai et al., 1999). A field experiment with increased precipitation and reduced wind velocity had been conducted since middle of April in 2016. Here, we investigated the effects of increased precipitation and reduced wind velocity on annual herb plant community structure and composition, in order to understand how the two factors and the interaction of those impact annual herb plant community characteristic, as well as how the species traits‐coverage, density, height, species diversity indexes, and ground biomass respond to the changing environment in the semiarid sandy grassland. We comprehensively proposed two hypotheses: (a) the increased precipitation and reduced wind velocity can stimulate the annual herb plant development (b) the interaction of both the two factors on plants community characteristics will be more complicated, possibly caused the larger changes in the annual herb plant community structure and composition.

## 2 MATERIALS AND METHODS

### 2.1 Site description

The study was conducted in Naiman Desertification Research Station (NDRS), Chinese Academy of Sciences, located in the south part of Horqin Sandy Land (42°58′N, 120°43′E; altitude approx. 360 m; Liu, He, Zhang, et al., 2015; Liu, He, Zhao, et al., 2015). The study area belongs to temperate semiarid continental and monsoonal climate. The long‐term mean annual precipitation is approximately 357 mm (Liu, He, Zhang, et al., 2015; Liu, He, Zhao, et al., 2015), this regional precipitation from May to August accounts for approximately 78.9% of the total amount. The mean annual potential evaporation is about 1,935 mm. The mean annual atmospheric wind velocity varies from 3.2 to 4.1 m/s, northwest wind in winter and spring, as well as southwest to south wind in summer and autumn prevailingly flow every year (Zuo et al., 2008, 2009). The soil in our studied region is classified as Cambic Arenosol (Su, Li, & Zhao, 2006). Sustained wind blowing easily erodes this regional soil. There are many native plants living in our experimental region, including *Salsola collina Pall*., *Bassia dasyphylla* (Fisch. & C. A. Mey.) *Kuntze*, *Setaria viridis* (L.) *Beauv*., *Corispermum macrocarpum Bunge var. macrocarpum*, *Cleistogenes squarrosa* (Trin.) *Keng*, *Caragana microphylla Lam*, *Lespedeza bicolor Turcz*.

### 2.2 Experiment design

**Figure 1:**
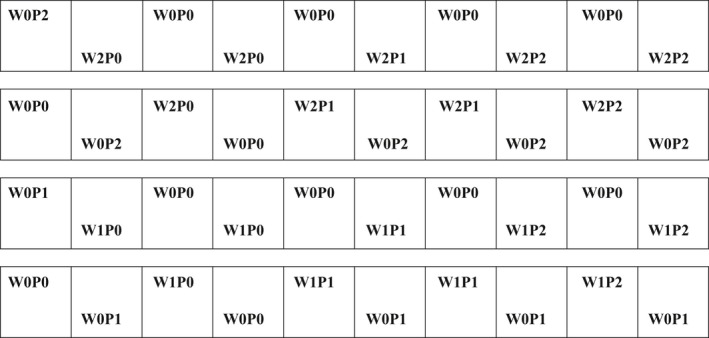
Schematic drawing of the study plots

Beginning from the middle of April, 2016, the experiment was conducted in forty concreted pouring pools of 2 m × 2 m, buried in 1 m depth under soil. This experiment collected 0–5 cm surface soil from fixed dune of six random locations, mixing it into soil package, then the soil passing through a 10 mm sieve to remove the debris. The sieved soil was homogeneously paved in forty concreted squared pools with same stratification and depth of the fixed dune. Soil seed bank mainly contains gramineous plants, which are dominant herbaceous plants in the fixed dune of Horqin Sandy Land, the seeds were randomly added into the forty concreted pouring pools on March 15th. Some seeds become dominant species due to the changing environmental factors, which is consistent with the dominant herbaceous plants in the fixed sand dunes under natural condition. There were three gradients in the precipitation and wind velocity treatment, respectively, with natural precipitation (P0), 30% increment in precipitation (P1), 60% increment in precipitation (P2); natural wind velocity (W0), 20% reduction in wind velocity (W1), and 40% reduction in wind velocity (W2). The process of adding water was instantly implemented at the end of the natural precipitation. Wrapped by one layer and two layers 2 m × 2 m × 1 m of 3 mm rectangle wind‐proof net, the W1 and W2 pools reduced wind velocity by 20% and 40% respectively, and the device of portable wind anemometer has monitored that the used wind‐proof net definitely reduced 20% and 40% natural wind in the corresponding pools (Table 1). Under the replicated and compared two factors of precipitation and wind velocity, there are nine treatments in our experiment: under the natural wind velocity, the natural precipitation (W0P0), the 30% increment in precipitation (W0P1) and the 60% increment in precipitation (W0P2), under the 20% reduction in wind velocity, the natural precipitation (W1P0), the 30% increment in precipitation (W1P1) and the 60% increment in precipitation (W1P2), under the 40% reduction in wind velocity, the natural precipitation (W2P0), the 30% increment in precipitation (W2P1) and the 60% increment in precipitation (W2P2), at least three replications for each treatment (Figure 1). The dominant plants in our experimental plots were *S. collina*, *B. dasyphylla*, *S. viridis*, *Corispermum elongatum*, and *Artemisia sieversiana Ehrh. ex Willd*.

**Table 1:** Soil temperature, air temperature, soil moisture, and wind velocity in experiment area

Experiment treatment	June				July				August			
	ST	AT	SM	WV	ST	AT	SM	WV	ST	AT	SM	WV
W0P0	21.89 ± 0.15	27.97 ± 0.25	5.79 ± 0.16	0.97 ± 0.03	24.28 ± 0.14	28.52 ± 0.20	7.21 ± 0.18	0.97 ± 0.03	23.85 ± 0.13	28.33 ± 0.28	7.68 ± 0.19	0.78 ± 0.02
W0P1	21.99 ± 0.23	27.13 ± 0.44	6.47 ± 0.28	1.11 ± 0.09	24.42 ± 0.20	28.52 ± 0.28	8.30 ± 0.35	1.08 ± 0.04	24.28 ± 0.22	28.20 ± 0.39	8.61 ± 0.30	0.86 ± 0.03
W0P2	21.35 ± 0.20	27.85 ± 0.33	6.83 ± 0.25	0.92 ± 0.03	23.73 ± 0.20	28.91 ± 0.30	7.69 ± 0.31	0.87 ± 0.03	23.81 ± 0.21	28.36 ± 0.41	8.00 ± 0.31	0.77 ± 0.02
W1P0	21.67 ± 0.40	26.93 ± 0.56	6.05 ± 0.27	0.75 ± 0.03	23.84 ± 0.25	27.40 ± 0.33	7.08 ± 0.33	0.77 ± 0.03	23.89 ± 0.24	28.32 ± 0.53	7.50 ± 0.33	0.65 ± 0.02
W1P1	21.53 ± 0.27	26.45 ± 0.54	6.72 ± 0.35	0.74 ± 0.03	23.84 ± 0.25	27.55 ± 0.37	8.31 ± 0.44	0.72 ± 0.02	23.99 ± 0.26	28.05 ± 0.51	8.52 ± 0.40	0.68 ± 0.02
W1P2	20.96 ± 0.25	26.64 ± 0.49	7.67 ± 0.36	0.72 ± 0.03	23.54 ± 0.25	27.26 ± 0.35	8.36 ± 0.44	0.70 ± 0.03	23.77 ± 0.26	27.83 ± 0.48	8.47 ± 0.42	0.63 ± 0.01
W2P0	20.79 ± 0.28	27.53 ± 0.58	6.17 ± 0.33	0.63 ± 0.01	23.01 ± 0.28	27.83 ± 0.40	7.37 ± 0.36	0.63 ± 0.01	23.04 ± 0.24	28.25 ± 0.53	7.83 ± 0.38	0.61 ± 0.01
W2P1	20.68 ± 0.25	27.42 ± 0.47	6.92 ± 0.28	0.64 ± 0.01	23.21 ± 0.26	27.75 ± 0.38	7.46 ± 0.35	0.63 ± 0.01	23.19 ± 0.25	28.38 ± 0.52	7.98 ± 0.39	0.60 ± 0.00
W2P2	20.68 ± 0.26	27.59 ± 0.55	7.15 ± 0.32	0.65 ± 0.01	23.08 ± 0.27	27.92 ± 0.37	7.74 ± 0.42	0.68 ± 0.02	23.00 ± 0.26	28.89 ± 0.51	8.49 ± 0.42	0.60 ± 0.00

Abbreviations: AT, air temperature; P0, natural precipitation; P1, the 30% increment in precipitation; P2, the 60% increment in precipitation; SM, soil moisture; ST, soil temperature; W0, natural wind velocity; W1, the 20% reduction in wind velocity; W2, the 40% reduction in wind velocity; WV, wind velocity.

### 2.3 Vegetation and microenvironment measurements

**Figure 2:**
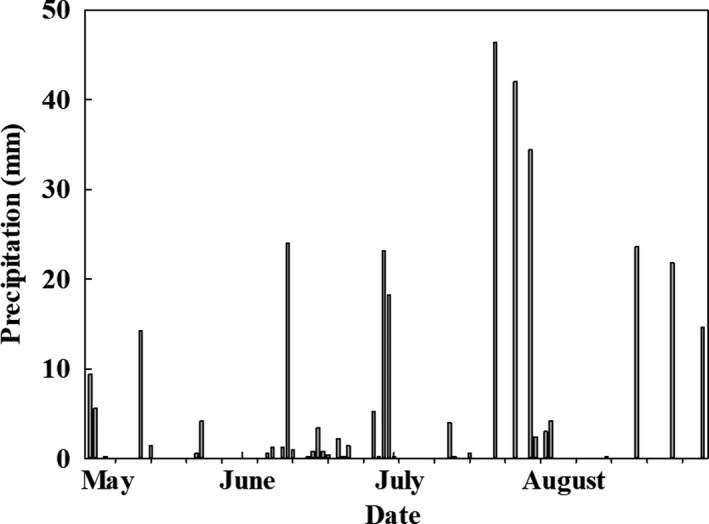
Precipitation in growing season (from May to August)

From May to August, we monitored precipitation, wind velocity (WV), air temperature (AT), soil moisture (SM) and soil temperature (ST), and investigated monthly vegetation characteristic. The examined results of wind velocity and soil moisture corresponded to our experimental design, lower wind velocity with reduced wind velocity plots, higher soil moisture with increased precipitation (Table 1). In our experiment, the most proportion of precipitation was falling in June and July (Figure 2). The precipitation was immediately measured at the end of it using three standard rainfall cylinders, set around the experiment plots. The wind velocity was monitored at the peak plant canopy by a portable wind anemometer three times a day starting at 8:00 a.m., internal 4 hr. Soil moisture and soil temperature in the soil depths in 0–20 cm of 40 pools were recorded using a portable soil moisture device (IMKO, TRIME‐Probe P3S) and digital probe thermometer (DT‐131) every day at the same time 8:00 a.m. We investigated vegetation characteristics once or twice a month using a 1 m × 1 m frame with 100 equally distributed grids (10 cm × 10 cm), randomly putting above the canopy in each squared pool, on June 16th, the middle of June (M‐June), July 4th, the beginning of July (B‐July), July 26th, the end of July (E‐July) and August 15th, the middle of August (M‐August). In our experiment, we define the number of the species types and the single species frequency as species richness and species abundance, respectively; we used the total of all the abundance of species as the functional group abundance (Klanderud & Totland, 2005). We randomly measured the canopy height of sixteen plants of the same species and calculated their average value as the species height.

### 2.4 Data analysis

We processed the experimental variables and significance test through the software of SPSS (version 19.0). We pretested the experimental data for the homogeneity of variance. One‐way ANOVA (analysis of variance) was performed to test the individual effect of increased precipitation and reduced wind velocity on species coverage, density and height, species diversity index, and ground biomass of the annual herb plant community. The interactive effect between precipitation increase and wind reduction on the same above parameters of annual herb plant was tested using the two‐way ANOVA. The experimental results were checked through LSD's test (*p* < .05). We calculated the species important value (SIV) using the parameters of relative abundance (RA), relative height (RH), and relative cover (RC) of the species: SIV=(RA+RH+RC)/3. The species diversity indexes include species richness, Simpson index, Shannon–Wiener index and Pielou Evenness index (Zhang, Zhao, Zhang, Zhao, & Drake, 2005; Zuo et al., 2008).

## 3 RESULTS

### 3.1 Annual herb plant traits

**Figure 3:**
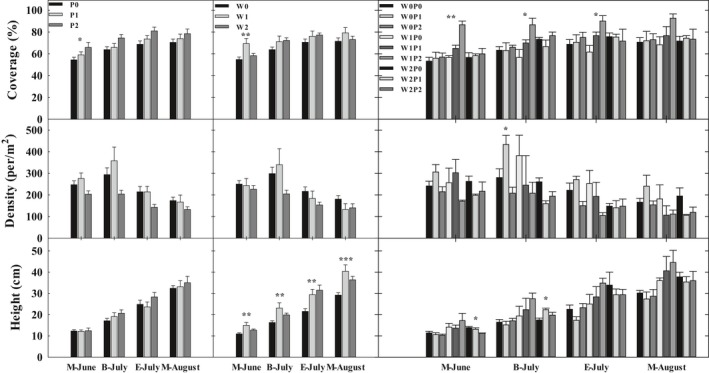
Annual herb plant community traits under precipitation and wind velocity treatment. M‐June: June 16th; B‐July: July 4th; E‐July: July 26th; M‐August: August 15th. Significance level: *p < .05; **p < .01; ***p < .001

The increased precipitation and reduced wind velocity stimulated coverage of annual herb plant community in the growing season (Figure 3), and there is a significant augment especially in M‐June with increased precipitation (*p* < .05) and reduced wind velocity (*p* < .01). Moreover, the height of annual herb plant under reduced wind velocity was significantly higher than that under the natural wind in M‐June, B‐July, E‐July and M‐August. The 20% reduction in wind velocity caused 36.67%, 41.60%, 36.62%, and 38.49% larger height compared with that under natural wind in M‐June, B‐July, E‐July, and M‐August, respectively.

There was obvious increment in the herb coverage and height with the interaction of the 20% reduced wind velocity and precipitation treatment in M‐June, B‐July, E‐July, and M‐August. Under the same 60% increased precipitation, the reduced wind velocity stimulated the herb coverage and height, the 20% reduced wind caused 52.05%, 31.32%, 20.00%, 26.95% higher coverage and elevated 66.90%, 61.04%, 49.73%, 55.34% larger height than that under natural wind velocity in M‐June, B‐July, E‐July, and M‐August, respectively.

**Figure 4:**
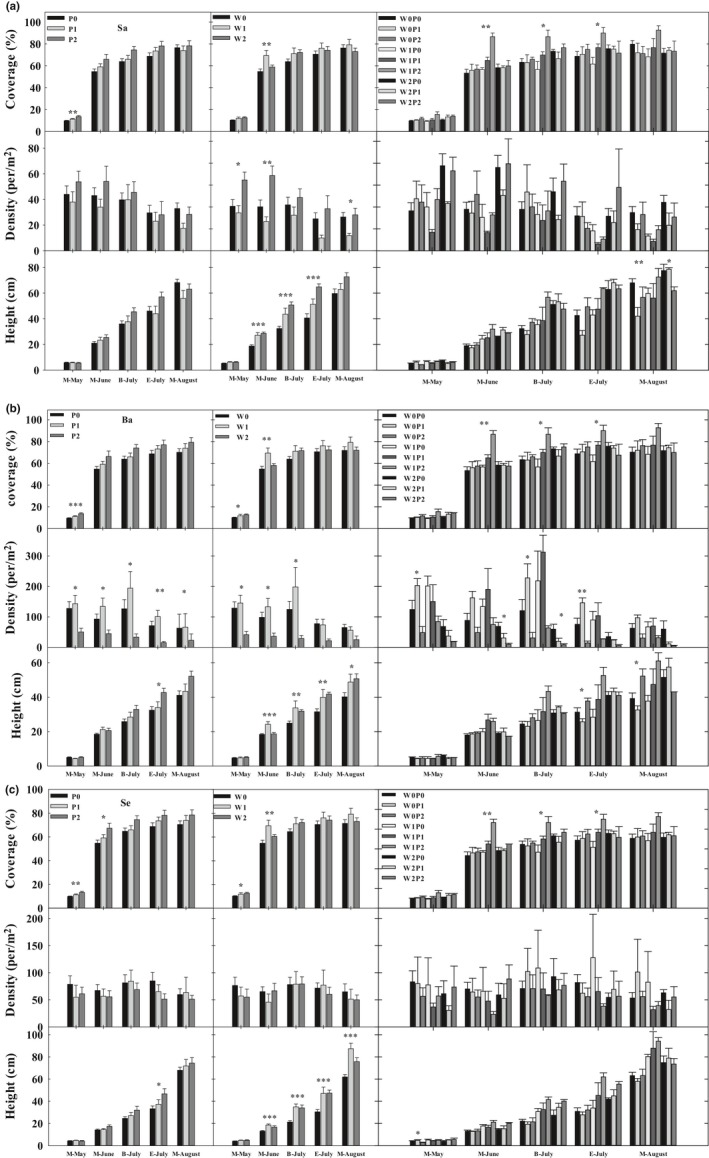
Dominant plant traits under precipitation and wind velocity treatment. Ba, *Bassia dasyphylla*; Sa, *Salsola collina*; Se, *Setaria viridis*. Significance level: *p < .05; **p < .01; ***p < .001

The increased precipitation and reduced wind velocity stimulated the coverage and height of the dominant plants, *S. collina*, *B. dasyphylla*, and *S. viridis*, from May to August (Figure 4). The 60% increment in precipitation generally caused the more considerable elevation of the three dominant herbs’ coverage and height compared with the 30% increment in precipitation from May to August. Moreover, the 40% reduction in wind velocity elevated *S. collina*'s larger height than that in the 20% reduced wind from May to August, but the 20% reduced wind velocity caused the larger height of *B. dasyphylla* and *S. viridis* in M‐May, M‐June, and B‐July. Furthermore, there was a fluctuation in the three dominant herb's density with increased precipitation and reduced wind velocity. The interaction of the 60% increment in precipitation and the 20% reduction in wind velocity significantly stimulated the largest coverage and height of the three dominant herbs from May to August.

### 3.2 Annual herb plant species diversity index

**Figure 5:**
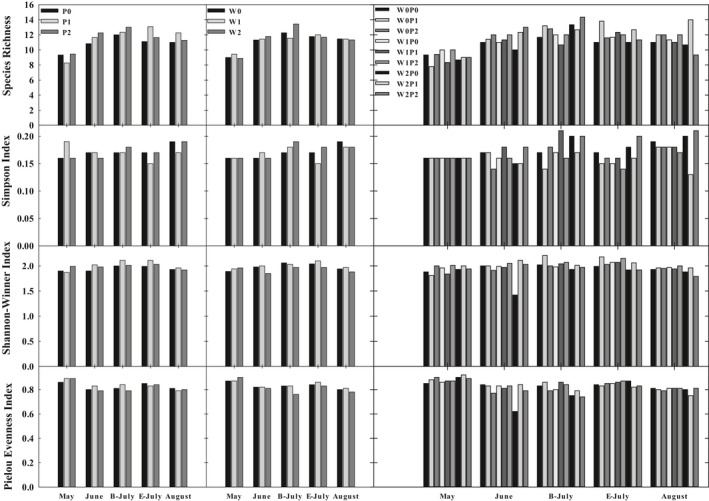
Sandy annual herb plant community species diversity in precipitation and wind velocity treatment. B‐July: beginning of July, July 4th; E‐July: ending of July, July 26th

The increased precipitation elevated the species richness and Shannon–Wiener index of annual herb plant in June and July (Figure 5). The reduced wind velocity, as well as the interaction of both increased precipitation and reduced wind velocity caused a fluctuation in species diversity indexes of annual herb plant.

### 3.3 Annual herb plant biomass

**Figure 6:**
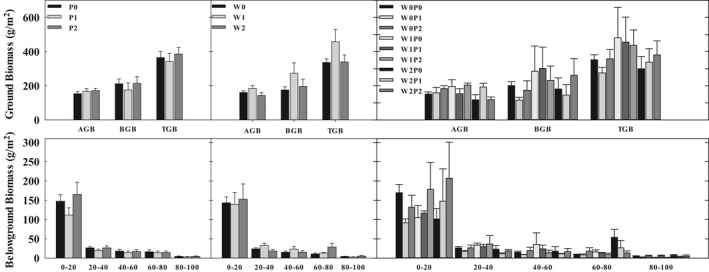
Distribution in sandy annual herb plant community aboveground and belowground biomass with increased precipitation and reduced wind velocity. AGB, aboveground biomass; BGB, belowground biomass; TGB, total ground biomass

The aboveground biomass and belowground biomass could be stimulated by the increased precipitation and reduced wind velocity, respectively (Figure 6). Moreover, the 20% reduction in wind velocity considerably elevated the ground biomass. Furthermore, the 60% increased precipitation, as well as the 40% reduced wind velocity caused increment of belowground biomass in 0–20 cm depth.

## 4 DISCUSSION

In arid and semiarid sandy grassland, water deficiency will significantly limit the plant development and productivity (Liu, Zhang, & Wan, 2009), precipitation magnitude is important to regulate the responding of plant community characteristics to changing environment (Dukes et al., 2005; Yang et al., 2011, 2015; Knapp, Ciais, & Smith, 2016). In our experiments, the increased precipitation considerably stimulates the height and coverage of the herbaceous community as well as the three dominant herbs. This result is positively related to increased precipitation magnitude significantly stimulating coverage of plant community in a temperate steppe (Yang et al., 2011, 2015). This result illustrates that the growing mechanism of annual herbaceous community is in consistent with increased precipitation, suggesting that annual herb community is relatively well‐adapted to the changing environment, which will make an important donation to maintain the stable ecosystem in sandy grassland plant community. Moreover, we found that wind flowing plays a critical role in controlling the herbaceous height, the decreased wind flowing will elevate the plant community height in grassland. This finding also shows that the interactive mechanism of precipitation and wind flowing is much more complicated than the single treatment. Their interaction could utmost promote the annual herb plant community growth since the interaction of 60% increment in precipitation and 20% reduction in wind velocity led to the largest plant coverage and height during the growing season.

The responses of different plant functional groups to increased precipitation and reduced wind velocity changed the annual herb community composition in the sandy grassland. The growing characteristic of annual herbaceous dominant species *S. collina*, *B. dasyphylla*, and *S. viridis* influenced by changing precipitation and wind velocity. In our experimental results, the growing mechanism of dominant species was in agreement with that of the regional annual herb community. The density changing mechanism of the annual herb community and the three dominant plants was not clear detected, but we have examined the largest and lowest dominant plants' density in the beginning of July and the middle of August, respectively, with the continuously growing coverage. We analyzed this phenomenon through the possible explanations: The dominant species in our study, *S. collina*, *B. dasyphylla*, and *S. viridis* stopped growing then died in the end of July, or the continuously growing canopy intensified interspecific competition, suppressed the growth of subordinated species on the experiment plots. Higher *S. collina* plant and bigger canopy of *B. dasyphylla* are likely to be exposed to more light in our pools and tend to benefit more from the increased precipitation and lower wind, the low‐stature plants or subdominant plants are consequently suppressed to extend their height and expand their coverage (Yang et al., 2015), which implied that interspecific competition pattern may be regulated by the changing environment, and we should take species interactions into consideration while evaluating plant community response to the global changing climate.

Our results demonstrated that precipitation increase significantly promotes species richness in this semiarid sandy land during the growing season, identical to the experimental results conducting in the annual grassland (Zavaleta et al., 2003) and the mesic habitat (Stevens, Shirk, & Steiner, 2006) as well as the temperate steppe of northern China (Yang et al., 2011, 2015). Water availability could regulate the plant germination, establishment and development rate, with further effects on the species richness in the experiment areas (Stevens et al., 2006). We need to conduct longer‐term experiment to clear explain how the changing precipitation mediate the community structure (Kardol et al., 2010), for instance, how the variation of species richness connects with the changing soil moisture (Yang et al., 2011), which was not detected in our results. We acknowledge that the nutrient increase is likely to suppress the species richness (Stevens et al., 2006), given that reduced wind velocity declines nutrient in our experiment (Sydeman et al., 2014), the interactive effect between increased precipitation and reduced wind velocity apparently stimulates species richness during the growing season, this result is consistent with our assumption. Furthermore, there are few changes in species diversity of annual herbs, suggesting that the annual herb community structure is relatively stable and successfully adapted to the changing environment.

Precipitation increase could enhance the plant biomass and productivity (Dukes et al., 2005; Wu et al., 2011). Some researchers have illustrated that changing climatic factors could interact or superpose to impact plant biomass (Kardol et al., 2010; Shaw et al., 2002), correspondingly, we found that the interaction between precipitation and wind velocity stimulated plant aboveground biomass in our experiment. We also found that water availability had a larger impact on aboveground biomass compared with the atmospheric wind, and the precipitation did not interact with atmospheric wind to universally accumulate biomass. This result suggests that the interaction of precipitation and atmospheric wind is likely to regulate the relationship between plant and water, then alleviating the influence of water‐limitation on plant biomass production (Kardol et al., 2010; Luo et al., 2010).

## 5 CONCLUSIONS

Our study shows that supplement precipitation evidently enhances plant species richness, in consistent with previous study of increased precipitation impacting mechanism. Increased precipitation, lower wind velocity as well as their interaction significantly stimulate plant development. Our study indicates that water availability dominates the increment of plant coverage, while wind activity made primary donation to plant height elevation. Our findings also suggest the positive relation of the dominant plants to the whole community ecosystem, and researcher should take more attention to the dominant species prospering. Furthermore, the study has important implication for understanding and predicting herbaceous plant community characteristics and mechanism of the semiarid ecosystems under changing climatic environment.

## CONFLICT OF INTEREST

None declared.

## AUTHOR CONTRIBUTIONS

Shanshan Sun designed the research, collected and analyzed the data and wrote the paper. Xinping Liu and Yuhui He designed the research and assisted with paper revision. Shuilian Wei and Lamei Zhang conducted the experiment, collected the data and did some laboratory analysis. Peng Lv and Chelmeg collected and analyzed the data, assisted with paper revision. Mingming Wang and Li Cheng assisted with data collection and did some laboratory analysis.

## Data Availability

My Dryad account is Ssshan93@163.com (or Sun). I will archive my data to the Dryad and complete the data accessibility statement, including database and information.
